# Association between Chronic Obstructive Pulmonary Disease and Ménière’s Disease: A Nested Case—Control Study Using a National Health Screening Cohort

**DOI:** 10.3390/ijerph18094536

**Published:** 2021-04-24

**Authors:** So-Young Kim, Chang-Ho Lee, Dae-Myoung Yoo, Chan-Yang Min, Hyo-Geun Choi

**Affiliations:** 1 CHA Bundang Medical Center, Department of Otorhinolaryngology-Head & Neck Surgery, CHA University, Seongnam 13496, Korea; sossi81@hanmail.net (S.-Y.K.); hearwell@gmail.com (C.-H.L.); 2 Hallym Data Science Laboratory, Hallym University College of Medicine, Anyang 14068, Korea; ydm1285@naver.com (D.-M.Y.); joicemin@naver.com (C.-Y.M.); 3 Graduate School of Public Health, Seoul National University, Seoul 08826, Korea; 4 Department of Otorhinolaryngology-Head & Neck Surgery, Hallym University College of Medicine, Anyang 14068, Korea

**Keywords:** Ménière’s disease, chronic obstructive pulmonary disease, risk factors, case–control studies, cohort studies

## Abstract

This study explored the relation between Ménière’s disease and chronic obstructive pulmonary disease (COPD). The ≥40-year-old population of the Korean National Health Insurance Service-Health Screening Cohort was included. In total, 7734 Ménière’s disease patients and 30,936 control participants were enrolled. Control participants were matched for age, sex, income, and region of residence with Ménière’s disease participants. The odds of having Ménière’s disease given a history of COPD were analyzed using conditional logistic regression. Subgroup analyses were conducted according to age, sex, income, and region of residence. The odds of having Ménière’s disease were found to be 1.18-fold higher with a history of COPD than with no history of COPD (95% confidence intervals (CI) = 1.06–1.32, E-value (CI) = 1.64 (1.31)). The ≥60 years old, male, low-income, and rural subgroups showed increased odds of developing Ménière’s disease when a history of COPD was reported. A history of COPD was associated with an increased risk of Ménière’s disease in the adult population.

## 1. Introduction

Chronic obstructive pulmonary disease (COPD) is a chronic obstructive airway disease with a prevalence of approximately 10.1% worldwide [1]. COPD is more common in men than in women, and mortality due to COPD occurs mainly in low- to middle-income countries [2]. COPD is ranked as having the third highest burden for a disease of mortality worldwide [3]. Furthermore, with an aging population, the disease burden of COPD has been increasing [4]. Although COPD is a heterogeneous and complex disease [5], progressive airway inflammation and an impaired immune response are common pathologies in patients with COPD [6,7]. COPD shares risk factors, including aging, smoking, inflammation, and physical inactivity, with other comorbidities, such as cardiovascular disease, osteoporosis, and depression [8]. In addition, COPD has been suggested to increase the risk of these comorbidities via aggravation of inflammatory and immune responses and physical inactivity [7].

Ménière’s disease is described as episodic cochleovestibular symptoms of vertigo and fluctuating hearing loss [9]. Because the diagnosis of Ménière’s disease depends on the symptom presentations, the early stage of Ménière’s disease is difficult to identify [10]. The prevalence of Ménière’s disease has been reported to be approximately 17–46 per 100,000 people [11,12]. The incidence of Ménière’s disease is high in female and middle-aged populations [11,12]. Endolymphatic hydrops has been acknowledged as the primary histopathology of Ménière’s disease [13]. The obstruction and/or overflow of endolymphatic flow could cause Ménière’s disease [13]. The etiology of Ménière’s disease has been suggested to be multifactorial, including a genetic predisposition for autoantigens and immune problems [14]. COPD has also been suggested to be caused by autoimmune problems [15]. In addition, many COPD patients suffer from neurological symptoms, such as dizziness and depression, although these symptoms are often ignored due to their chronic comorbid status. A previous study suggested the impact of chronic hypoxemia in COPD patients on cochleovestibular dysfunction [16]. Abnormalities in otoacoustic emission, hearing threshold level, and latencies of auditory brainstem response were higher in the COPD groups than in the control group [16].

The hypothesis of the present study was that COPD may be associated with the high occurrence of Ménière’s disease. To test this hypothesis, we recruited patients with Ménière’s disease from the national health claim database, and those with a history of COPD were compared with subjects in the control group. There has been a lack of evidence for the association of COPD with Ménière’s disease. Therefore, the E-value was calculated to estimate the possible effect of unmeasured confounders [17].

## 2. Materials and Methods

### 2.1. Study Population

This study was approved by the ethics committee of Hallym University (2019-10-023). Because this study used retrospective health claims data, the requirement for written informed consent was waived by the ethics committee of Hallym University. The guidelines and regulations of the ethics committee of Hallym University were followed during all analyses. The Korean National Health Insurance Service-Health Screening Cohort data were used [18].

### 2.2. Participant Selection

A total of 9032 patients with Ménière’s disease were selected from among 514,866 patients with 615,488,428 medical claim codes. The exclusion criteria were as follows: a diagnosis of Ménière’s disease from 2002 to 2003 (*n* = 963); a history of treatment for head trauma (S00-S09, diagnosed by neurologists, neurosurgeons, or emergency medicine doctors) with head and neck CT evaluations (*n* = 275); treatment for brain tumors (C70–C72, *n* = 14), disorders of acoustic nerves (H933, *n* = 22), or benign neoplasms of cranial nerves (D333, *n* = 23); or patients missing total cholesterol level measurements (*n* = 1).

A total of 505,834 control participants who were not included in the Ménière’s disease group from 2002 to 2015 were randomly selected. The exclusion criteria were as follows: patients who died before 2004; patients with no records since 2004; patients treated for Ménière’s disease (ICD-10 codes: H810) without an audiometric examination (*n* = 16,549); patients with a history of treatment for head trauma with head and neck CT evaluations (*n* = 12,607); and patients treated for brain tumors (C70–C72, *n* = 820), acoustic nerve disorders (H933, *n* = 120), and benign neoplasms of cranial nerves (D333, *n* = 191).

**Figure 1:**
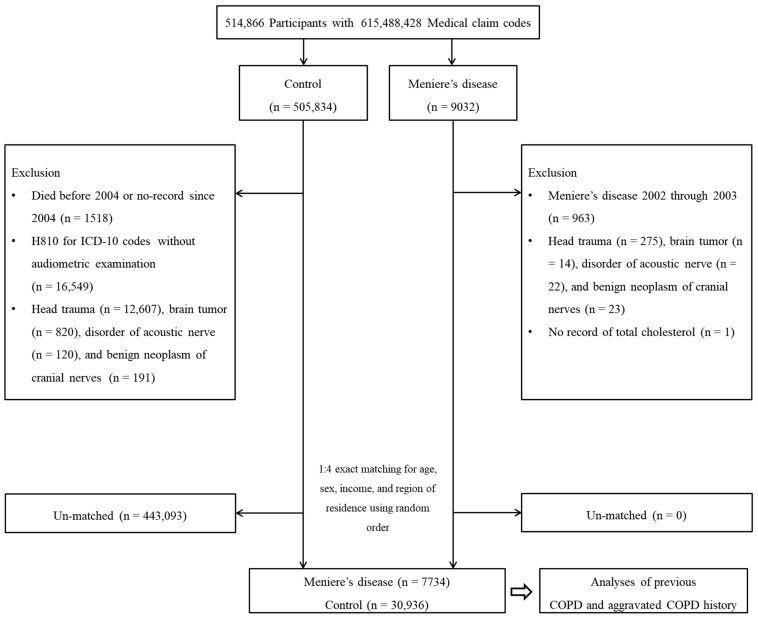
A schematic illustration of the participant selection process that was used in the present study. Of a total of 514,866 participants, 6054 Ménière’s disease participants were matched with 24,216 control participants for age, sex, income, and region of residence. Abbreviations: COPD, chronic obstructive pulmonary disease.

The control subjects were matched 1:4 with the Ménière’s disease patients for age, sex, income, and region of residence. The diagnosis date of Ménière’s disease was set as the index date. Finally, 7734 Ménière’s disease participants and 30,936 control participants were enrolled (Figure 1).

### 2.3. Independent Variables (Chronic Obstructive Pulmonary Disease and Aggravation)

Chronic obstructive pulmonary disease was defined as the occurrence of unspecified chronic bronchitis (J42), emphysema (J43), or other COPD (J44), except MacLeod syndrome (J430) ≥2 times with COPD-related medications prescribed ≥2 times, including long-acting muscarinic antagonists (LAMAs), long-acting β2-agonists (LABAs), inhaled corticosteroids (ICSs) combined with LABAs, short-acting muscarinic antagonists (SAMAs), short-acting β2 agonists (SABAs), methylxanthine, PDE4 inhibitors, and systemic beta agonists [19].

COPD aggravation was defined as a visit to the emergency room or admission to the hospital with a COPD diagnosis.

### 2.4. Dependent Variable (Ménière’s Disease)

Ménière’s disease was included based on a history of ≥2 incidents of treatment for Ménière’s disease (H810) and a previous audiometric examination (claim code: E6931–E6937, F6341–F6348) [20].

### 2.5. Covariates

The covariates included age group, income level, region of residence, obesity based on body mass index (BMI (kg/m^2^)), tobacco smoking (nonsmoker, past smoker, or current smoker), and alcohol consumption (<one time a week or ≥one time a week), which were classified as described in our previous studies [21,22]. Additional measurement data, namely, total cholesterol level (mg/dL), systolic blood pressure (SBP, mmHg), diastolic blood pressure (DBP, mmHg), and fasting blood glucose level (mg/dL), were included.

Asthma was diagnosed based on a history of ≥2 incidents of treatment for asthma (J45, J46) and a history of ≥2 prescriptions for asthma medications, including ICSs or ICSs combined with LABAs, oral leukotriene antagonists (LTRAs), SABAs, systemic LABAs, xanthine derivatives, or systemic corticosteroids [21,23].

The covariates of benign paroxysmal vertigo (H811, ≥2 times), vestibular neuronitis (H812, ≥2 times), and other peripheral vertigo (H813, ≥2 times) were diagnosed based on the diagnostic codes (ICD-10). To assess the burden of comorbidities, the Charlson Comorbidity Index (CCI), except for pulmonary disease, was calculated as the continuous variable (0 (no comorbidities) to 29 (multiple comorbidities)) [24,25].

### 2.6. Statistical Analyses

The general characteristics were compared between the Ménière’s disease and control groups using the chi-square test for categorical variables and the Wilcoxon rank-sum test for continuous variables.

Conditional logistic regression stratified by age, sex, income, and region of residence was conducted to estimate the odds ratios (ORs) and 95% confidence intervals (CIs) of COPD for Ménière’s disease. The analysis was adjusted for the following covariates: asthma, obesity, smoking, alcohol consumption, CCI scores, total cholesterol level, SBP, DBP, fasting blood glucose level, benign paroxysmal vertigo, vestibular neuronitis, and other peripheral vertigo.

Subgroup analyses were conducted for age (<60 years old and ≥60 years old), sex (males and females), income (low income and high income), and region of residence (urban and rural). Additional analyses were performed in subgroups according to obesity, smoking, alcohol consumption, total cholesterol levels, blood pressure, fasting blood glucose levels, and CCI score. In these subgroups, Model 1 (adjusted for age, sex, income, and region of residence) and Model 2 (model 1 plus obesity, smoking, alcohol consumption, CCI scores, total cholesterol level, SBP, DBP, fasting blood glucose level, benign paroxysmal vertigo, vestibular neuronitis, other peripheral vertigo, and asthma) were analyzed using unconditional logistic regression.

The COPD group was divided into aggravated and non-aggravated COPD groups, and the OR of COPD for Ménière’s disease was analyzed.

E-values were calculated as the sensitivity analyses [26]. SAS version 9.4 (SAS Institute Inc., Cary, NC, USA) was used for statistical analysis. All analyses were two-tailed. A *p* value < 0.05 was considered statistically significant.

## 3. Results

A total of 8.5% (660/7734) and 6.2% (1922/30,936) of Ménière’s disease and control participants had histories of COPD, respectively (*p* < 0.001, Table 1). There were significant differences in the prevalence of obesity, smoking status, alcohol consumption, and CCI scores between the patients with Ménière’s disease and the control group (all *p* < 0.001).

A history of COPD was associated with increased odds of having Ménière’s disease (adjusted OR (aOR) = 1.18, 95% CI = 1.06–1.32, *p* = 0.004, E-value (CI) = 1.64 (1.31), Table 2). The positive relation of Ménière’s disease with COPD was persistent in the subgroups based on age, sex, income, and region of residence (all *p* < 0.05). The aORs of a history of COPD for Ménière’s disease were 1.18 (95%CI = 1.05–1.33, E-value (CI) = 1.64 (1.28)) in the ≥60 years old group, 1.28 (95%CI = 1.07–1.52, E-value (CI) = 1.88 (1.34)) in the male group, 1.26 (95%CI = 1.07–1.48, E-value (CI) = 1.83 (1.34)) in the low-income group, and 1.19 (95%CI = 1.04–1.37, E-value (CI) = 1.67 (1.24)) in the rural group. 

Analysis of other subgroups based on obesity, smoking, alcohol consumption, total cholesterol level, blood pressure, fasting blood glucose level, and CCI score also showed an association of COPD with higher odds of having Ménière’s disease (Table S1). Notably, however, there was no significant difference in the odds of having Ménière’s disease between the patients with aggravated COPD and those with non-aggravated COPD (Table 3). None of the age, sex, income, or region of residence subgroups showed a definitive relation between aggravation of COPD and Ménière’s disease.

## 4. Discussion

COPD was associated with an increased risk of Ménière’s disease in the adult population. COPD patients showed 18% higher odds for Ménière’s disease than that of the control group after adjusting for lifestyle factors of obesity, smoking, alcohol consumption, past medical histories, and laboratory findings. Other vestibular disorders of benign paroxysmal vertigo, vestibular neuronitis, and other peripheral vertigo were also adjusted. These associations were also valid in the over 60, male, low-income, and rural subgroups. The present findings proposed a novel link between COPD and Ménière’s disease. Although the pathophysiology of these links between COPD and Ménière’s disease has not been described, a few possible pathophysiological factors can be explained.

Autoimmunity, as a shared pathophysiology, could mediate the association of COPD with Ménière’s disease. Autoimmunity has been reported to be related to Ménière’s disease [27]. Approximately one-third of cases of Ménière’s disease were attributed to autoimmune origin [27]. Autoimmunization to type II collagen induced endolymphatic hydrops in a guinea pig model [28]. In addition, autoantibodies against inner ear antigens were found in the sera of Ménière’s disease patients [29]. Autoimmunity has also been proposed as one of the pathophysiologies of COPD [30]. The imbalance between pulmonary matrix proteases and antiproteases, which may be influenced by the generation of adaptive immune and autoimmune responses, could lead to the development of COPD [30]. The accumulation of proinflammatory T helper (Th) 1 and Th17 cells, which resemble the autoimmune response, and impaired regulatory T cell functions were observed in COPD patients [15]. In addition, autoantibodies are present in the blood of COPD patients, and autoimmunity against pulmonary antigens was observed in COPD animal models [31]. Because Ménière’s disease could be diagnosed after the onset of recurrent cochleovestibular symptoms, COPD can manifest before Ménière’s disease.

Ischemia may mediate the association of COPD with Ménière’s disease. Insufficient inner ear blood supply has been noted as one of the pathophysiologies of inner ear dysfunction, including noise-induced hearing loss and Ménière’s disease [32,33,34]. Because the main generator of endolymph of the cochlea involves the stria vascularis, ischemic injuries to the inner ear could impair the endolymphatic flow in the inner ear [33]. Vascular abnormalities in the lateral wall of the cochlear duct are associated with endolymphatic hydrops in guinea pigs and humans [35]. In a cadaver study, Ménière’s disease patients showed occlusion of the vein of the vestibular aqueduct [36]. Notably, COPD may cause chronic ischemic status due to insufficient respiration and hypoxia. A number of previous studies demonstrated an association of COPD with myocardial infarction and stroke [37,38]. Thus, ischemia in COPD patients could hinder endolymphatic flow and induce Ménière’s disease. Although the exacerbation of COPD was associated with an increased risk of myocardial infarction or stroke in previous studies, there was no association between the aggravation of COPD and Ménière’s disease in the present study [37,39]. However, the relatively small size of the population with aggravated COPD (*n* = 124) could limit the statistical power in the present results. In addition, the high morbid state due to the aggravated COPD could deter the diagnosis of Ménière’s disease. 

In the subgroup analysis, COPD was associated with Ménière’s disease in the elderly, male, low-income, and rural subgroups in this study. The high prevalence of COPD among elderly individuals could mediate the higher relevance of COPD to Ménière’s disease [40]. In addition, the vulnerability to comorbidities due to age-related changes may influence the strong association of COPD with Ménière’s disease in the elderly population. The phenotypes, symptoms, and comorbidities of COPD have been reported to show sex differences [41]. Although the incidence of COPD has shown an increasing tendency in women, men have a higher rate of COPD [41]. These sex differences could impact the association of COPD with Ménière’s disease. Low socioeconomic status was also acknowledged as a risk factor for COPD [42]. Low annual income and rented housing were associated with 2.23 (95% CI = 1.97–2.53) and 1.41 (95% CI = 1.30–1.52) times higher risks of COPD, respectively [42]. The increased susceptibility to COPD may be linked with the association between COPD and Ménière’s disease in these poor populations.

This study first verified the association of COPD with Ménière’s disease. The large representative population provided a large control population. However, due to the health claim code database, laboratory findings, such as pulmonary function tests and vestibular function tests, were not available. The difficulty of diagnosis of Ménière’s disease could have originated from the combined cochleovestibular dysfunction in as high as 71.5% of moderate to profound sensorineural hearing loss patients [43]. In addition, anatomical variation could trigger Menieriform syndrome [44]. The heterogeneous severity and types of COPD and Ménière’s disease could have underestimated the association of COPD with Ménière’s disease. The present study considered the lifestyle factors of smoking, obesity, and alcohol consumption. However, potential confounders, including stress level, that were not included might have influenced the current results.

## 5. Conclusions

A history of COPD could be a risk factor for the occurrence of Ménière’s disease in the adult population. The association of COPD with Ménière’s disease was solid in vulnerable populations, such as over 60, male, low-income, and rural subgroups. Clinicians should consider the co-occurrence of Ménière’s disease in COPD patients who suffer from dizziness or hearing discomfort.

## Figures and Tables

**Table 1:** General characteristics of participants.

Characteristics	Total Participants		
	Ménière’s Disease	Control	*p*-Value
Age (years old, *n*, %)			1.000
40–44	72 (0.9)	288 (0.9)	
45–49	470 (6.1)	1880 (6.1)	
50–54	1130 (14.6)	4520 (14.6)	
55–59	1336 (17.3)	5344 (17.3)	
60–64	1257 (16.3)	5028 (16.3)	
65–69	1231 (15.9)	4924 (15.9)	
70–74	1115 (14.4)	4460 (14.4)	
75–79	726 (9.4)	2904 (9.4)	
80–84	318 (4.1)	1272 (4.1)	
85+	79 (1.0)	316 (1.0)	
Sex (n, %)			1.000
Males	2752 (35.6)	11,008 (35.6)	
Females	4982 (64.4)	19,928 (64.4)	
Income (n, %)			1.000
1 (lowest)	1343 (17.4)	5372 (17.4)	
2	967 (12.5)	3868 (12.5)	
3	1193 (15.4)	4772 (15.4)	
4	1605 (20.8)	6420 (20.8)	
5 (highest)	2626 (34.0)	10,504 (34.0)	
Region of residence (n, %)			1.000
Urban	3267 (42.2)	13,068 (42.2)	
Rural	4467 (57.8)	17,868 (57.8)	
Total cholesterol (mg/dL, mean, SD)	200.3 (38.3)	200.3 (38.4)	0.976
SBP (mmHg, mean, SD)	126.2 (16.2)	127.1 (16.9)	<0.001 ^†^
DBP (mmHg, mean, SD)	77.6 (10.3)	78.1 (10.6)	0.001 ^†^
Fasting blood glucose (mg/dL, mean, SD)	99.7 (25.3)	100.5 (28.2)	0.900
Obesity (n, %) ^‡^			<0.001 *
Underweight	152 (2.0)	823 (2.7)	
Normal	2638 (34.1)	10,997 (35.6)	
Overweight	2168 (28.0)	8305 (26.9)	
Obese I	2541 (32.9)	9803 (31.7)	
Obese II	235 (3.0)	1008 (3.3)	
Smoking status (n, %)			<0.001 *
Nonsmoker	6249 (80.8)	24,375 (78.8)	
Past smoker	813 (10.5)	3048 (9.9)	
Current smoker	672 (8.7)	3513 (11.4)	
Alcohol consumption (n, %)			<0.001 *
<1 time a week	5802 (75.0)	22,326 (72.2)	
≥1 time a week	1932 (25.0)	8610 (27.8)	
CCI score (score, n, %) ^§^			<0.001 *
0	5088 (65.8)	21,884 (70.7)	
1	1337 (17.3)	4023 (13.0)	
2	664 (8.6)	2420 (7.8)	
3	314 (4.1)	1135 (3.7)	
≥4	331 (4.3)	1474 (4.8)	
Benign paroxysmal vertigo (n, %)	2630 (34.0)	1915 (6.2)	<0.001 *
Vestibular neuronitis (n, %)	842 (10.9)	438 (1.4)	<0.001 *
Other peripheral vertigo (n, %)	1809 (23.4)	1390 (4.5)	<0.001 *
Asthma (n, %)	1839 (23.8)	5535 (17.9)	<0.001 *
COPD (n, %)	660 (8.5)	1922 (6.2)	<0.001 *
Non-aggravated COPD	536 (6.9)	1549 (5.0)	
Aggravated COPD	124 (1.6)	373 (1.2)	

Abbreviations: CCI, Charlson comorbidity index; COPD, chronic obstructive pulmonary disease; DBP, diastolic blood pressure; SBP, systolic blood pressure; SD, standard deviation. * Chi-square test. Significance at *p* < 0.05. ^†^ Wilcoxon rank-sum test. Significance at *p* < 0.05. ^‡^ Obesity (BMI, body mass index, kg/m^2^) was categorized as <18.5 (underweight), ≥18.5 to <23 (normal), ≥23 to <25 (overweight), ≥25 to <30 (obese I), and ≥30 (obese II). ^§^ CCI scores were calculated without pulmonary disease.

**Table 2:** Odds ratios (95% confidence intervals) of chronic obstructive pulmonary disease for Ménière’s disease with subgroup analyses according to age, sex, income, and region of residence.

Characteristics	No. of Ménière’s Disease	Odds Ratios for Ménière’s Disease				
	/No. of Participants (%)	Crude ^†^	*p*-Value	Adjusted ^†,‡^	*p*-Value	E-Value (CI)
Total participants (*n* = 38,670)						
COPD	660/2582 (25.6)	1.43 (1.30–1.57)	<0.001 *	1.18 (1.06–1.32)	0.004 *	1.64 (1.31)
Non-COPD	7074/36,088 (19.6)	1		1		
Age <60 years old (*n* = 15,040)						
COPD	107/381 (28.1)	1.59 (1.26–1.99)	<0.001 *	1.25 (0.95–1.63)	0.108	1.81 (1.00)
Non-COPD	2901/14,659 (19.8)	1		1		
Age ≥ 60 years old (*n* = 23,630)						
COPD	553/2201 (25.1)	1.40 (1.26–1.55)	<0.001 *	1.18 (1.05–1.33)	0.008 *	1.64 (1.28)
Non-COPD	4173/21,429 (19.5)	1		1		
Males (*n* = 13,760)						
COPD	296/1103 (26.8)	1.56 (1.35–1.80)	<0.001 *	1.28 (1.07–1.52)	0.006 *	1.88 (1.34)
Non-COPD	2456/12,657 (19.4)	1		1		
Females (*n* = 24,910)						
COPD	364/1479 (24.6)	1.34 (1.18–1.52)	<0.001 *	1.11 (0.96–1.28)	0.153	1.46 (1.00)
Non-COPD	4618/23,431 (19.7)	1		1		
Low income (*n* = 17,515)						
COPD	324/1214 (26.7)	1.53 (1.33–1.75)	<0.001 *	1.26 (1.07–1.48)	0.005 *	1.83 (1.34)
Non-COPD	3179/16,301 (19.5)	1		1		
High income (*n* = 21,155)						
COPD	336/1368 (24.6)	1.35 (1.18–1.53)	<0.001 *	1.11 (0.95–1.29)	0.202	1.46 (1.00)
Non-COPD	3895/19,787 (19.7)	1		1		
Urban (*n* = 16,335)						
COPD	229/901 (25.4)	1.41 (1.20–1.65)	<0.001 *	1.17 (0.97–1.41)	0.103	1.62 (1.00)
Non-COPD	3038/15,434 (19.7)	1		1		
Rural (*n* = 22,335)						
COPD	431/1681 (25.6)	1.44 (1.28–1.62)	<0.001 *	1.19 (1.04–1.37)	0.012 *	1.67 (1.24)
Non-COPD	4036/20,654 (19.5)	1		1		

Abbreviations: CCI, Charlson comorbidity index; CI, confidence interval; COPD, chronic obstructive pulmonary disease; DBP, diastolic blood pressure; SBP, systolic blood pressure. * Conditional logistic regression, significance at *p* < 0.05. ^†^ Models were stratified by age, sex, income, and region of residence. ^‡^ Adjusted for obesity, smoking, alcohol consumption, CCI scores, total cholesterol, SBP, DBP, fasting blood glucose, benign paroxysmal vertigo, vestibular neuronitis, other peripheral vertigo, and asthma.

**Table 3:** Odds ratios (95% confidence intervals) of aggravated chronic obstructive pulmonary disease for Ménière’s disease with subgroup analyses according to age, sex, income, and region of residence in COPD participants.

Characteristics	No. of Ménière’s Disease	Odds Ratios for Ménière’s Disease				E-Value
	/No. of Participants (%)	Model 1 ^†^	*p*-Value	Model 2 ^‡^	*p*-Value	Estimate (CI)
Total participants (*n* = 2582)						
Aggravated COPD	124/497 (25.0)	0.97 (0.76–1.22)	0.773	0.99 (0.76–1.29)	0.935	1.12 (1.00)
Non-aggravated COPD	536/2085 (25.7)	1		1		
Age <60 years old (*n* = 381)						
Aggravated COPD	8/31 (25.8)	0.88 (0.37–2.10)	0.774	0.75 (0.26–2.15)	0.587	2.02 (1.00)
Non-aggravated COPD	99/350 (28.3)	1		1		
Age ≥60 years old (*n* = 2201)						
Aggravated COPD	116/466 (24.9)	0.97 (0.76–1.24)	0.814	1.01 (0.76–1.33)	0.963	1.09 (1.00)
Non-aggravated COPD	437/1735 (25.2)	1		1		
Males (*n* = 1103)						
Aggravated COPD	84/304 (27.6)	1.07 (0.79–1.45)	0.655	1.19 (0.83–1.69)	0.352	1.65 (1.00)
Non-aggravated COPD	212/799 (26.5)	1		1		
Females (*n* = 1479)						
Aggravated COPD	40/193 (20.7)	0.82 (0.57–1.20)	0.312	0.77 (0.50–1.18)	0.226	1.93 (1.00)
Non-aggravated COPD	324/1286 (25.2)	1		1		
Low income (*n* = 1214)						
Aggravated COPD	59/225 (26.2)	1.04 (0.74–1.46)	0.831	0.98 (0.66–1.45)	0.907	1.18 (1.00)
Non-aggravated COPD	265/989 (26.8)	1		1		
High income (*n* = 1368)						
Aggravated COPD	65/272 (23.9)	0.92 (0.67–1.27)	0.604	1.04 (0.72–1.50)	0.827	1.25 (1.00)
Non-aggravated COPD	271/1096 (24.7)	1		1		
Urban (*n* = 901)						
Aggravated COPD	30/144 (20.8)	0.77 (0.49–1.21)	0.263	0.89 (0.53–1.49)	0.657	1.50 (1.00)
Non-aggravated COPD	199/757 (26.3)	1		1		
Rural (*n* = 1681)						
Aggravated COPD	94/353 (26.6)	1.05 (0.80–1.39)	0.706	1.06 (0.77–1.45)	0.737	1.30 (1.00)
Non-aggravated COPD	337/1328 (25.4)	1		1		

Abbreviations: CCI, Charlson comorbidity index; CI, confidence interval; COPD, chronic obstructive pulmonary disease; DBP, diastolic blood pressure; SBP, systolic blood pressure. Un-conditional logistic regression, significance at *p* < 0.05. ^†^ Model 1 was adjusted for by age, sex, income, and region of residence. ^‡^ Model 2 was adjusted for age, sex, income, region of residence, obesity, smoking, alcohol consumption, CCI scores, total cholesterol, SBP, DBP, fasting blood glucose, benign paroxysmal vertigo, vestibular neuronitis, other peripheral vertigo, and asthma.

## Data Availability

Release of the data by the authors is not legally allowed. All of the data are available on the database of the National Health Insurance Sharing Service (NHISS) https://nhiss.nhis.or.kr/ (accessed on 2 June 2019). NHISS permits access to all of these data via download at a certain cost for any researcher who promises to follow the research ethics.

## Supplementary Materials

The following are available online at https://www.mdpi.com/article/10.3390/ijerph18094536/s1, Table S1: Subgroup analyses of odds ratios (95% confidence intervals) of chronic obstructive pulmonary disease for Ménière’s disease according to obesity, smoking, alcohol consumption, total cholesterol, blood pressure, blood glucose, and Charlson comorbidity index.
