# Supplementary material for: Association between Chronic Obstructive Pulmonary Disease and Ménière’s Disease: A Nested Case—Control Study Using a National Health Screening Cohort
Source: Int J Environ Res Public Health. 2021 Apr 24;18(9):4536. doi: 10.3390/ijerph18094536 (PMC8123129; doi:10.3390/ijerph18094536)
Supplement: Supplementary file 1 [file ijerph-18-04536-s001.zip › ijerph-1192768-supplementary.pdf]

Table S1 Subgroup analyses of odds ratios (95% confidence intervals) of chronic obstructive pulmonary disease for Meniere's disease according to obesity, smoking, alcohol consumption, total cholesterol, blood pressure, blood glucose, and Charlson comorbidity index.

Characteristics	No. of Meniere's disease/ No. of participants (%)	Odds ratios for Meniere's disease			
		Model 1†	P-value	Model 2‡	P-value
Obesity (Underweight, n = 975)					
COPD	23/119 (19.3)	1.20 (0.72-1.99)	0.485	1.03 (0.56-1.88)	0.934
Non-COPD	129/856 (15.1)	1		1	
Obesity (Normal weight, n = 13,635)					
COPD	235/934 (25.2)	1.49 (1.27-1.74)	<0.001*	1.26 (1.05-1.52)	0.014*
Non-COPD	2,403/12,701 (18.9)	1		1	
Obesity (Overweight, n = 10,473)					
COPD	166/628 (26.4)	1.41 (1.17-1.70)	<0.001*	1.11 (0.89-1.39)	0.340
Non-COPD	2,002/9,845 (20.3)	1		1	
Obesity (Obese, n = 13,587)					
COPD	236/901 (26.2)	1.43 (1.22-1.67)	<0.001*	1.16 (0.97-1.40)	0.105

Non-COPD	2,540/12,686 (20.0)	1		1	
Smoking (Nonsmoker, n = 30,624)					
COPD	499/1,917 (26.0)	1.42 (1.27-1.58)	<0.001*	1.18 (1.04-1.33)	0.011*
Non-COPD	5,750/28,707 (20.0)	1		1	
Smoking (Past smoker and current smoker, n = 8,046)					
COPD	161/665 (24.2)	1.51 (1.25-1.83)	<0.001*	1.18 (0.93-1.49)	0.187
Non-COPD	1,324/7,381 (17.9)	1		1	
Alcohol consumption (< 1 time a week, n = 28,128)					
COPD	503/1,964 (25.6)	1.36 (1.23-1.52)	<0.001*	1.13 (0.99-1.28)	0.064
Non-COPD	5,299/26,164 (20.3)	1		1	
Alcohol consumption (≥ 1 time a week, n = 10,542)					
COPD	157/618 (25.4)	1.58 (1.31-1.92)	<0.001*	1.34 (1.07-1.68)	0.010*
Non-COPD	1,775/9,924 (17.9)	1		1	
Total cholesterol (< 200 mg/dL, n = 19,992)					
COPD	362/1,455 (24.9)	1.38 (1.21-1.57)	<0.001*	1.12 (0.97-1.31)	0.130
Non-COPD	3,627/18,537 (19.6)	1		1	

Total cholesterol (≥ 200 to < 240 mg/dL, n = 13,186)					
COPD	198/789 (25.1)	1.41 (1.19-1.67)	<0.001*	1.24 (1.02-1.51)	0.033*
Non-COPD	2,411/12,397 (19.5)	1		1	
Total cholesterol (≥ 240 mg/dL, n = 5,492)					
COPD	100/338 (29.6)	1.69 (1.32-2.17)	<0.001*	1.29 (0.96-1.73)	0.086
Non-COPD	1,036/5,154 (20.1)	1		1	
Blood pressure (SBP < 140 mmHg and DBP < 90 mmHg, n = 28,878)					
COPD	507/1,926 (26.3)	1.44 (1.29-1.60)	<0.001*	1.17 (1.03-1.33)	0.014*
Non-COPD	5,409/26,952 (20.1)	1		1	
Blood pressure (SBP ≥ 140 mmHg or DBP ≥ 90 mmHg, n = 9,792)					
COPD	153/656 (23.3)	1.35 (1.11-1.64)	0.002*	1.21 (0.96-1.51)	0.105
Non-COPD	1,665/9,136 (18.2)	1		1	
Fasting blood glucose (< 100 mg/dL, n = 24,421)					
COPD	398/1,599 (24.9)	1.35 (1.20-1.52)	<0.001*	1.16 (1.01-1.34)	0.036*
Non-COPD	4,499/22,822 (19.7)	1		1	
Fasting blood glucose (≥ 100 mg/dL, n = 14,249)					

COPD	262/983 (26.7)	1.55 (1.34-1.81)	<0.001*	1.20 (1.01-1.44)	0.043*
Non-COPD	2,575/13,266 (19.4)	1		1	
CCI score (score = 0, n = 25,975)					
COPD	263/1,110 (23.7)	1.36 (1.18-1.57)	<0.001*	1.12 (0.95-1.32)	0.194
Non-COPD	4,613/24,865 (18.6)	1		1	
CCI score (score = 1, n = 5,844)					
COPD	174/642 (27.1)	1.30 (1.07-1.57)	0.007*	1.26 (1.00-1.58)	0.047*
Non-COPD	1,238/5,202 (23.8)	1		1	
CCI score (score ≥ 2, n = 6,851)					
COPD	223/830 (26.9)	1.49 (1.26-1.77)	<0.001*	1.21 (0.98-1.48)	0.072
Non-COPD	1,223/6,021 (20.3)	1		1	

Abbreviations: CCI, Charlson comorbidity index; COPD, chronic obstructive pulmonary disease; DBP, diastolic blood pressure; SBP, systolic blood pressure

* Logistic regression, Significance at $P < 0.05$

† A model 1 was adjusted for age, sex, income, and region of residence.

‡ A model 2 was adjusted for age, sex, income, and region of residence, obesity, smoking, alcohol consumption, CCI scores, total cholesterol, SBP, DBP, fasting blood glucose, benign paroxysmal vertigo, vestibular neuronitis, other peripheral vertigo, and asthma
